# Third-generation sequencing identified a novel complex variant in a patient with rare alpha-thalassemia

**DOI:** 10.1186/s12887-024-04811-1

**Published:** 2024-05-13

**Authors:** Cong Zhou, Yepei Du, Haixia Zhang, Xing Wei, Rui Li, Jing Wang

**Affiliations:** 1 Department of Medical Genetics/Prenatal Diagnostic Center, Key Laboratory of Birth Defects and Related Diseases of Women and Children of MOE, State Key Laboratory of Biotherapy, West China Second University Hospital, Sichuan University, Chengdu, China; 2 Department of Medical Genetics/Prenatal Diagnostic Center, West China Second University Hospital, Sichuan University, Chengdu, Sichuan China; 3 Key Laboratory of Birth Defects and Related Diseases of Women and Children (Sichuan University), Ministry of Education, Chengdu, China; 4 Department of Biotherapy, Cancer Center and State Key Laboratory of Biotherapy, West China Hospital, Sichuan University, Chengdu, Sichuan China

**Keywords:** Alpha-thalassemia, Third-generation sequencing (TGS), Complex variant, Fertility risk

## Abstract

**Background:**

Thalassemias represent some of the most common monogenic diseases worldwide and are caused by variations in human hemoglobin genes which disrupt the balance of synthesis between the alpha and beta globin chains. Thalassemia gene detection technology is the gold standard to achieve accurate detection of thalassemia, but in clinical practice, most of the tests are only for common genotypes, which can easily lead to missing or misdiagnosis of rare thalassemia genotypes.

**Case presentation:**

We present the case of an 18-year-old Chinese female with abnormal values of routine hematological indices who was admitted for genetic screening for thalassemia. Genomic DNA was extracted and used for the genetic assays. Gap polymerase chain reaction and agarose gel electrophoresis were performed to detect *HBA *gene deletions, while PCR-reverse dot blot hybridization was used to detect point mutations in the *HBA* and *HBB* genes. Next-generation sequencing and third-generation sequencing (TGS) were used to identify known and potentially novel genotypes of thalassemia. We identified a novel complex variant α^Hb Westmead^α^Hb Westmead^α^anti3.7^/-α^3.7^ in a patient with rare alpha-thalassemia.

**Conclusions:**

Our study identified a novel complex variant that expands the thalassemia gene variants spectrum. Meanwhile, the study suggests that TGS could effectively improve the specificity of thalassemia gene detection, and has promising potential for the discovery of novel thalassemia genotypes, which could also improve the accuracy of genetic counseling. Couples who are thalassemia carriers have the opportunity to reduce their risk of having a child with thalassemia.

**Supplementary Information:**

The online version contains supplementary material available at 10.1186/s12887-024-04811-1.

## Background

Thalassemia is an autosomal recessive disease and one of the most common monogenic diseases worldwide [1]. Thalassemia is a hemoglobinopathy caused by variations (including mutations, deletions, duplications, and gene rearrangements) in alpha (α)- and beta (β)-globin gene clusters that disrupt the balance of synthesis between the α- and β-globin chains which compose hemoglobin [2]. The α-globin gene cluster lies within chromosome 16 [3]. The *HBA* locus contains two almost identical genes, *HBA1* and *HBA2*, which encode the α-hemoglobin chain [3, 4]. The clinical presentations of α-thalassemia vary widely [5, 6]. There are three types of α-thalassemia carrier states (silent, mild, and intermedia) and one disease state (major), depending on the number of globin chains and the disease state [7].

Approximately 5% and 1.5% of the global population carry mutations in α- and β-thalassemia genes, respectively [4, 8]. As a result, a large number of children are born each year with hemoglobin disorders which can lead to serious birth defects and place a heavy burden on society and families [9]. According to literature reports, the frequency of thalassemia gene carriage in southern China was 3–24%, the genotype of α^Hb Westmead^α/αα carrying rate was 3.41% [10] in Guangxi, China, and the prevalence of triplicated alpha thalassemia was 1.99% in Guangdong and Hunan province of China [11, 12]. Thus, accurate diagnosis of thalassemia in patients and carriers remains challenging owing to the complexity of thalassemia genetics and genotype-phenotype correlations.

Traditional DNA analysis methods for the diagnosis of thalassemia include gap polymerase chain reaction (Gap PCR), PCR reverse dot blot (PCR-RDB) hybridization, multiple ligation-dependent probe amplification, and Sanger sequencing [13, 14]. More recently, next-generation sequencing (NGS) methods have been used for genetic screening for thalassemia [15, 16]. In addition to discovering novel gene variants, these methods can effectively detect genotypes [16, 17]. In recent years, third-generation sequencing (TGS) technologies have been applied to detect thalassemia genes [18–20]. These technologies can generate ultra-long reads and achieve high sequence precision and are characterized by the absence of GC preference and single-molecule resolution [18, 19]. Such methods are helpful for the accurate diagnosis and subsequent treatment of disease and minimize the risk of missed diagnosis [18–20]. This study utilized TGS to identify a novel genotype of α-thalassemia.

## Case presentation

### Patient

An 18-year-old Chinese female sought genetic counseling from the Department of Medical Genetics of West China Second University Hospital of Sichuan University (Chengdu, China). She informed the doctor that she had an incomplete mediastinal uterus and primary infertility, and hematological examinations showed abnormal values of routine hematological indices: a mean cell hemoglobin (MCH) of 25 pg (reference: 27–31 pg) and a mean cell volume (MCV) of 78.2 fl (reference: 79–101 fl). The result of Hemoglobin (Hb) analysis was normal. Currently, the patient has no other abnormal clinical manifestations. The patient’s parents are healthy and there is no evidence of consanguineous marriage. The patient was admitted for genetic screening for thalassemia. This study was approved by the Medical Ethics Committee of West China Second University Hospital of Sichuan University, and written informed consent was obtained from the patient.

### Methods

#### DNA extraction

Blood samples were drawn from the patient after obtaining informed consent. Genomic DNA (gDNA) was extracted from samples using the QIAamp DNA Blood Mini Kit (Qiagen Bioinformatics, Hilden, Germany), according to the manufacturer’s instructions.

#### GAP-PCR testing for large-deletion α-thalassemia

Single-tube multiplex Gap-PCR was performed for the three common α-thalassemia deletions, including Southeast Asia (--^SEA^), rightward (- α^3.7^), and leftward (-α^4.2^), according to the manufacturer’s protocol (Yaneng Bioscience, Shenzhen, China).

#### PCR-RDB assays

PCR-RDB assay was performed for three common non-deletional α-thalassemia mutations in the *HBA1* and *HBA2* genes: c.427 T > C (Hb Constant Spring), c.377 T > C (Hb Quong Sze), and c.369 C > G (Hb Westmead). 17 β-thalassemia variations in the *HBB* gene were also screened for: c. -82 C > A [nt32(C→A)], c. -80 T > C [nt30(T→C)], c. -79 A > G [nt29(A→G)], c. -78 A > G [nt28(A→G)], c. -11_ -8delAAAC (CAP [UTR + 40-43(-AAAC)]), c.2 T > G [Int(ATG→AGG)], c.45_46insG [CD14-15(+ G)], c.52 A > T [CD17(A→T)], c.79G > A [βE(GAG→AAG)], c.84_85insC [CD27/28(+ C)], c.94delC [CD31(-C)], c.126_129delCTTT [CD41-42(-TTCT)], c.130G > T [CD43(G→T)], c.216_217insA [CD71-72(+ A)], c.92 + 1G > T [IVS-I-1(G→T)], c.92 + 5G > C [IVS-I-5(G→C)], and c.316-197 C > T [IVS-II-654(C→T)] (Yaneng Bioscience, Shenzhen, China).

#### Targeted NGS and data analysis

The M228 Kit (targeting 11 genes: *HBA1*, *HBA2*, *HBB*, *DMD*, *SMN1*, *GJB2*, *GJB3*, *SLC26A4*, *MT-RNR1*, *G6PD*, *ATP7B*) (MyGenostics, Inc. Beijing, China) was used to capture the targeted gDNA of patient according to the manufacturer’s instructions. The Nextseq CN500 mid Output Kit and NextSeq500 platform (Illumina, Inc. California, United States) was used to generate double-end sequencing reads (150 bp). The reads were mapped to GRCh37/hg19 reference by Burrows-Wheeler Aligner [21]. Variants were called using the Genome Analysis Tool Kit [22] and annotated by Annovar [23]. Then, all variants were filtered based on the frequency in the control population database (1,000 Genomes Project, ExAC, gnomad, Esp6500). Variants with a minimum allele frequency < 0.05 were retained. Several variant prediction tools have been used to predict the functional impact of candidate variants.

#### TGS and data analysis

The experiments were conducted as previously described [20]. gDNA was amplified using PCR with primers targeting the majority of known structural variation regions, single nucleotide variations (SNVs), and insertions and deletions (indels) in the *HBA1*, *HBA2*, and *HBB* genes. The PCR products were ligated to barcoded adaptors and ligation reactions to construct individual sequencing libraries were conducted. The libraries were quantified and pooled together by equal quality and converted to an Single molecule real-time (SMRT) bell library using the Sequel Binding and Internal Ctrl Kit 3.0 (Pacific Biosciences). The SMRT bell library was then sequenced in the CCS mode on the Sequel II platform (Pacific Biosciences) to generate 10–25 subreads per molecule. After sequencing, raw subreads were analyzed using CCS software (Pacific Biosciences) to generate CCS reads which were debarcoded by lima in the Pbbioconda package (Pacific Biosciences). After the alignment of the processed reads to genome build hg38 using pbmn2 (Pacific Biosciences), structural variations were identified according to the HbVar, Ithanet, and LOVD databases. The SNVs and indels were identified using FreeBayes1.3.4 (https://www.geneious.com/plugins/freebayes; Biomatters, Inc., San Diego, CA).

#### Variant confirmation by sanger sequencing

Sanger sequencing was performed on the gDNA of the patient to confirm SNVs. The primers were designed for standard polymerase chain reaction assays using Primer five software. The forward 5’-TTCTGGTCCCCACAGACTCA-3’ and reverse 5’-CAAAGACCAGGAAGGGCCG-3’ primer pairs were used to amplify Hb Westmead (c.369 of *HBA2*, reference transcript: NM_000517.6). Chromas software version 2.4.1 (Technelysium Pty Ltd., South Brisbane, Australia) was used to analyze the Sanger sequencing data.

#### Confirmation the ααα ^anti3.7^ by PCR-electrophoresis assay

The ααα ^anti3.7^ was verified using the PCR-electrophoresis according to the manufacturer’s protocol (six α-thalassemia gene detection kit, including HKαα, --^THAI^, fusion gene, ααα ^anti3.7^, ααα ^anti4.2^,-α^27.6^,Yaneng, Shenzhen, China).

### Clinical report

**Fig. 1:**
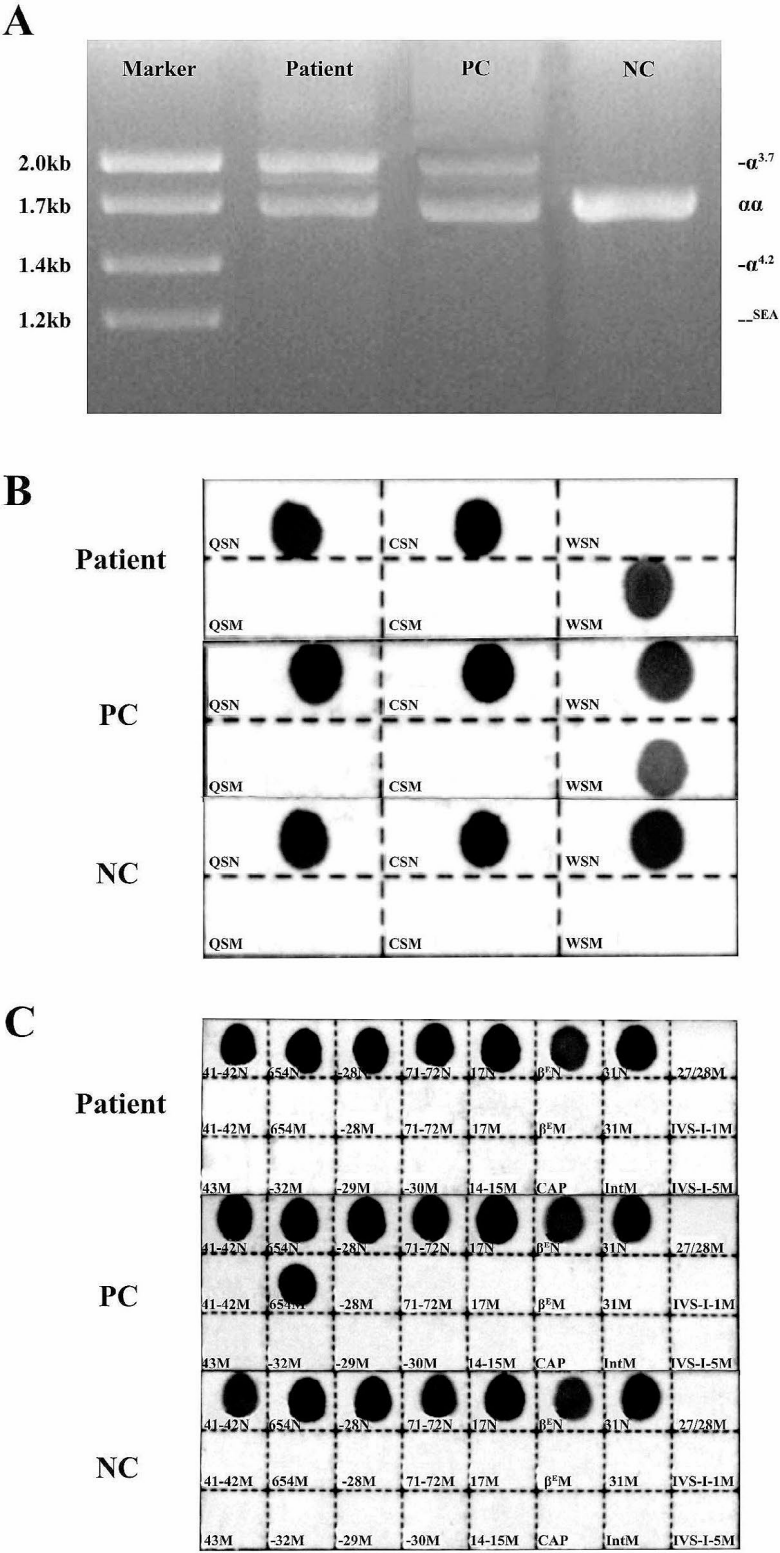
Routine genetic assays for 23 types of variations in *HBA* and *HBB* genes. (**A**) gap polymerase chain reaction (GAP PCR) was performed to detect 3 types of common deletions in a-thalassemia. The patient carries the -α^3.7^ heterozygous deletion. The positive control (PC) was -α^3.7^ heterozygosity deletion. (**B**) PCR reverse dot blot (PCR-RDB) assay was used to detect 3 types of common variants in a-thalassemia. The patient carries Hb Westmead homozygous variation. PC was Hb Westmead heterozygosity variation. (**C**) PCR-RDB assay was used to detect 17 types of common variation in β-thalassemia. The patient carries no variant of this type. PC was c.316-197 C > T (β^IVS−II−654M^) heterozygous variation (NC: Negative Control)

**Fig. 2:**
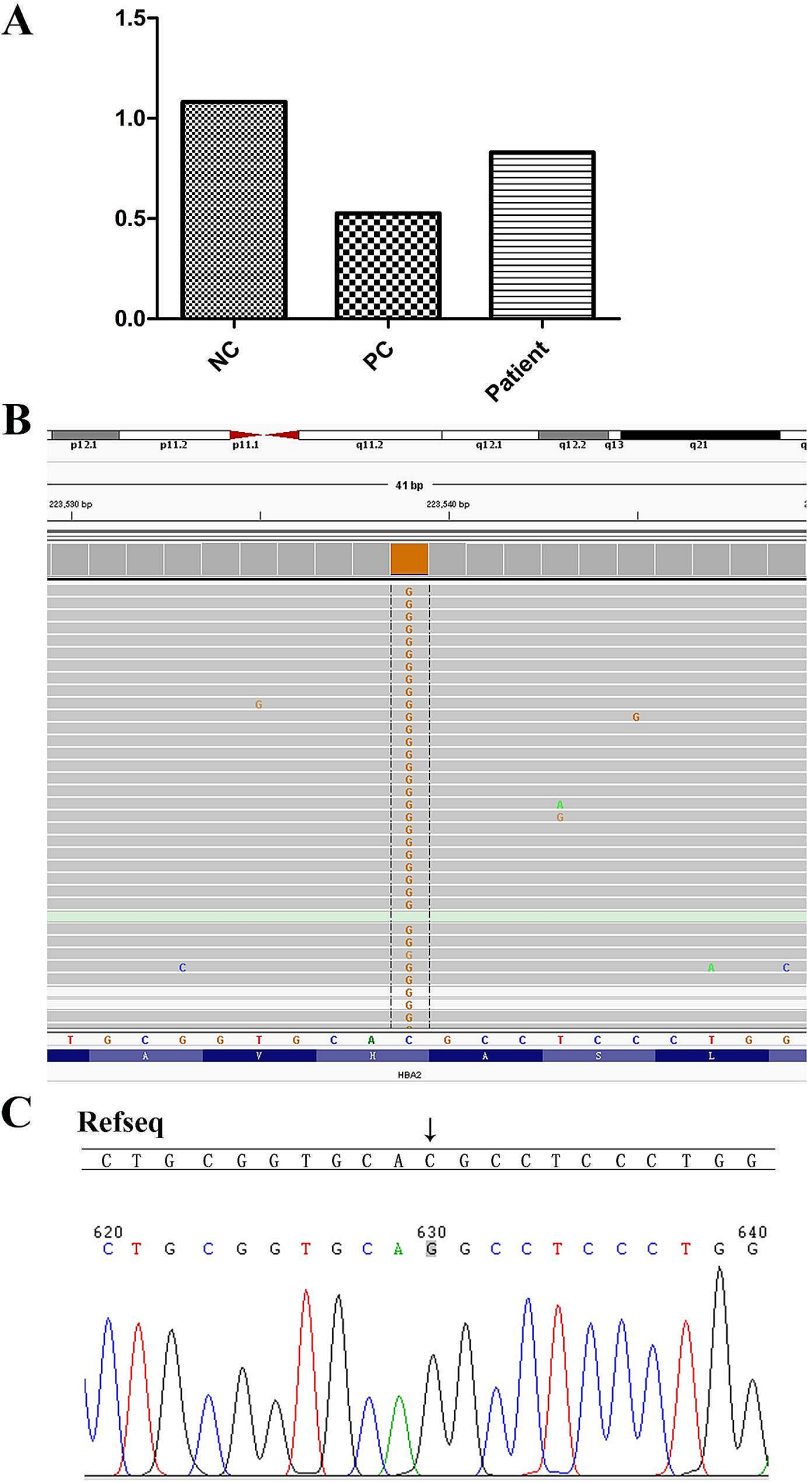
The results of targeted next-generation sequencing (NGS) and Sanger sequencing. (**A**) The copy number of α^3.7^ following the targeted NGS. The copy number ratio of α^3.7^ was 0.8 (between normal: 1 and loss of heterozygosity: 0.5). Positive control presented only a single copy and negative control showed a copy number of two (PC: positive control, NC: Negative control). (**B**) NGS results showed c.369 C > G homozygous variation. (**C**) Confirmation of the variant (c.369 C > G, homozygous) by Sanger sequencing

**Fig. 3:**
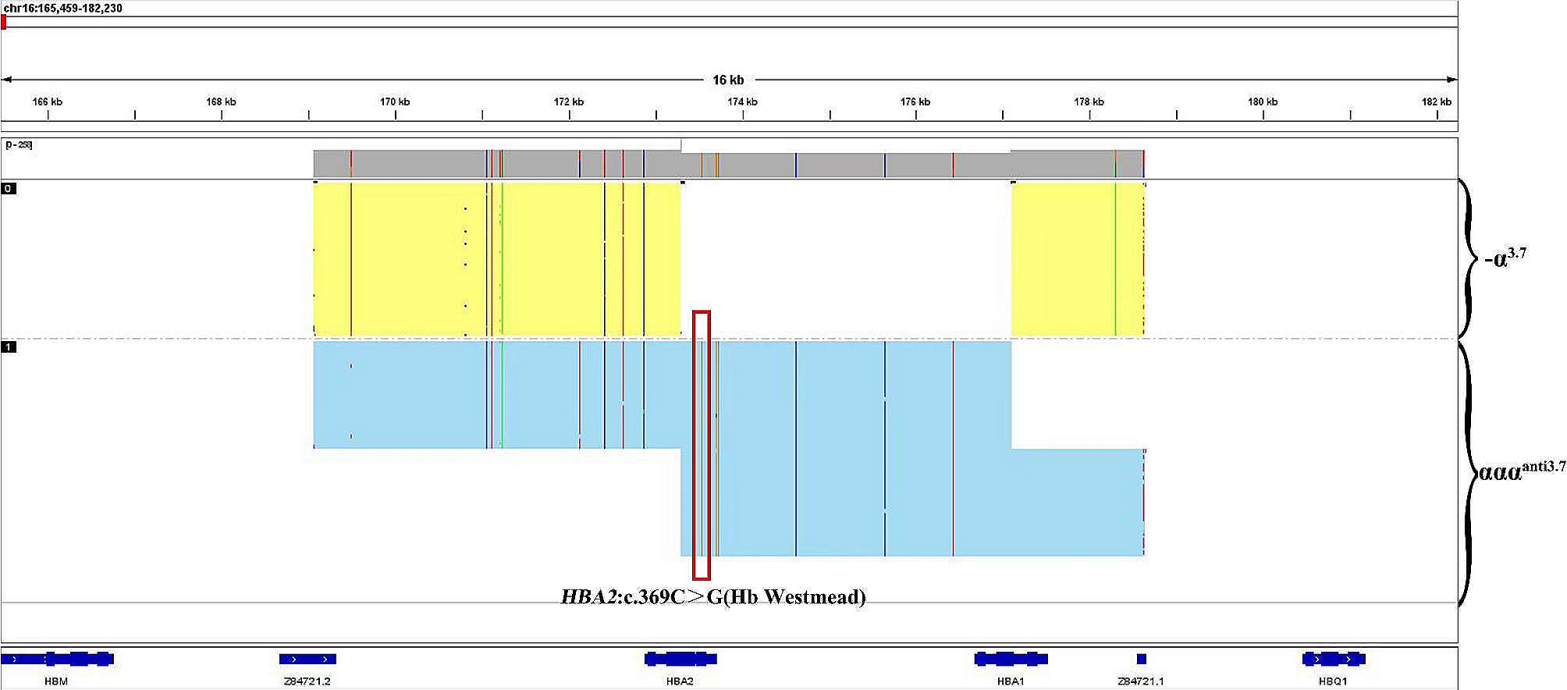
Third-generation sequencing showed one chromosome carried the -α^3.7^ deletion, and the other carried the ααα^anti3.7^. Two copies of the triplet carried the c.369 C > G variant

Gap PCR and PCR-RDB detected 23 genotypes in the most common thalassemia: three types of deletions (--^SEA^, -α^3.7^, -α^4.2^), three types of point mutations in *HBA* genes (α^Hb Constant Spring^α, α^Hb Quong Sze^α, and α^Hb Westmead^α), and 17 types of point mutations in the *HBB* gene. The results showed that the patient carried a -α^3.7^ heterozygous deletion and Hb Westmead (c.369 C > G) homozygous variation (Fig. 1). However, targeted NGS (sample was tested three times) showed that the copy number ratio of α^3.7^ was 0.8 (intermediate between normal, 1, and loss of heterozygosity, 0.5) (Fig. 2A). We also identified a homozygous variant (c.369 C > G) in the *HBA2* gene (NM_000517.6) (Fig. 2B). Sanger sequencing confirmed the targeted NGS results (Fig. 2C). Considering the results of NGS and large-deletion α-thalassemias were inconsistent, TGS was used. Interestingly, TGS analysis showed that one chromosome carried the -α^3.7^ deletion, while the other carried the ααα^anti3.7^ (confirmed using PCR-electrophoresis assay [24], Figure S1), two copies of the triplet which carried the *HBA2*: c.369 C > G (Hb Westmead) mutation (Fig. 3). To the best of our knowledge, this was the first report of the interaction between-3.7 Kb deletion (-α3.7 deletion) and triplicated alpha (anti-3.7) with homozygous Hb Westmead on two copies.

## Discussion and conclusion

The copy number of *HBA* genes (3, 2, 1, or none) in Asian patients with thalassemia results in four different α-thalassemia syndromes [25]. Three normal *HBA* genes result in a silent carrier state, usually without clinical symptoms [1, 2, 25]. Two normal *HBA* genes result in mild α-thalassemia, usually with Hypochromic microcytosis but presenting as asymptomatic, without anemia [1, 2, 4, 25]. One normal *HBA* gene results in Hb H disease, in which the clinical manifestations and the degree of anemia vary greatly. Patients with mild symptoms only had mild thalassemia without obvious clinical symptoms. In severe cases, regular blood transfusion is required, and obvious thalassemia features such as hepatosplenomegaly, thalassemia-like skeletal changes, jaundice, and others are present [1, 2, 4, 25]. The absence of a normal *HBA* gene results in homozygous α-thalassemia, which manifests as fatal hydrops fetalis. Hemoglobin (Hb) Bart’s edematous fetus is characterized by severe anemia, jaundice, systemic edema, hepatosplenomegaly, dysplasia, short limbs, and giant placenta, among other features, and is a fatal blood disease. The affected fetus usually dies in utero at 23–40 weeks of gestation or within half an hour after birth as a result of severe anemia and hypoxia [1–4, 25, 26].

Molecular diagnosis of thalassemia carrier states is challenging, especially because of the complexity of the *HBA* gene [9, 15]. Most thalassemia diagnostic laboratories use gap PCR and PCR-RDB to identify the most prevalent pathogenic *HBA*1/2 and *HBB* variants [6, 15]. These methods are mainly used to detect four common α-thalassemia deletions (--^SEA^, -α^3.7^, -α^4.2^, --^Thai^), three non-deletion α-thalassemia variants (α^Hb Constant Spring^α, α^Hb Quong Sze^α, and α^Hb Westmead^α), and 17 common β-thalassemia variants in the Chinese population [1, 15], and are economical and practical testing methods. Nevertheless, in addition to the 23 common thalassemia variants, there are hundreds of rare and emerging genotypes. In these cases, gap PCR and PCR-RDB cannot meet the detection requirements, and more accurate and efficient screening programs are needed. Recently, NGS has emerged as an alternative molecular method for the genetic detection of thalassemia. This method has the advantages of simple sample collection and highly accurate results [15–17]. Compared to traditional diagnostic methods, NGS can generate a large amount of genomic data and provide abundant genetic information. However, it may not be able to detect polystructures, tandem repeats, GC-rich regions, and other special structural regions, as well as highly homologous sequences [14, 15]. TGS, also called SMRT, was developed and validated using PacBio Sequel II [18]. Unlike NGS, which targets only exonic regions or selected intronic regions, TGS is used to generate longer PCR fragments that include both intergenic and intragenic regions improving the ability to identify variants [27–31]. Each DNA molecule is sequenced separately in TGS [18, 19]. TGS has multiple advantages including extremely long reads and being PCR-free [18–20]. Recently, TGS technology has become popular for the genetic detection of thalassemia [27–29]. TGS can detect all mutation types of α-thalassemia and β-thalassemia genes [18, 30, 31]. However, TGS is currently not widely used in clinical testing owing to its high cost.

In this study, different methods were used for screening thalassemia. First, the routine hematological phenotypes of the patient were detected, finding both MCV and MCH showed a mild decrease. Subsequent analysis of 23 common thalassemia variations showed that the patient carried the -α^3.7^ heterozygous deletion and Hb Westmead (c.369 C > G) homozygous variation. In our study, targeted NGS showed that the patient carried the homozygous variant c.369 C > G in *HBA2*, but the copy number of α^3.7^ was between normal, 1, and loss of heterozygosity, 0.5, which cannot be determined accurately. Finally, TGS analysis showed a novel genotype in the α-globin gene cluster: one chromosome carried the -α^3.7^ deletion, and the other carried the ααα^anti3.7^, with two copies of the triplet carrying the *HBA2*: c.369 C > G (Hb Westmead) variant. Therefore, the intermediate copy number identified through NGS can be understood in the context of the TGS results. The TGS results also intuitively showed that the Hb Westmead homozygous variation detected by PCR-RDB was not from the chromosome with the -α^3.7^ deletion. Compared with GAP-PCR, NGS has a wider detection range and can detect deletion types, point mutations, and other rare variants in common thalassemia screening. However, owing to the limitation of NGS testing over the highly homologous regions of *HBA1* and *HBA2* genes, there may be false positives and false negatives in the detection of α-deletion thalassemia. It is necessary to use other methods, such as TGS, for verification and typing. TGS uses long reads that could cover many rare gene loci, and its PCR-free characteristic means it is possible to reflect real arrangements in the genome [30, 31]. To the best of our knowledge, the genotypes identified in this study have not been reported previously. The genotype -α^3.7^/ααα^anti3.7^ has been reported in neonates from Mazandaran; however, the phenotype of the newborn has not been specifically described [32]. There are many types of variation in thalassemia-related genes, some of which are complex. When the clinical phenotype is inconsistent with the laboratory molecular test results, TGS should be performed to help accurately identify genetic variations. Accurate genetic test results are a prerequisite for the accurate assessment of reproductive risk.

In summary, a novel genotype in the α-globin gene cluster was confirmed by TGS in a Chinese female with mild decreases in MCV and MCH. Our study showed that TGS technology has the potential to detect novel variants which may be beyond the scope of traditional analytical methods. Therefore, TGS can be an effective and reliable approach for thalassemia screening in individuals suspected to carry rare mutations or complex variants. In addition, TGS analysis should be considered for the accurate diagnosis of uncertain cases of thalassemia, which could also improve the accuracy of genetic counseling. Couples who are thalassemia carriers have the opportunity to seek prenatal diagnosis and even preimplantation genetic testing services to reduce their risk of having a child with thalassemia.

### Electronic supplementary material

Below is the link to the electronic supplementary material.

Supplementary Material 1

## Data Availability

For the considerations about the security of human genetic resources and the confidentiality of participant, the data is not publicly available, but can be applied from the corresponding author on reasonable request.

## Abbreviations

TGS: Third-generation sequencing
Hb: Hemoglobin
α: Alpha
β: Beta
Gap PCR: Gap polymerase chain reaction
PCR-RDB: PCR reverse dot blot
SMRT: Single molecule real-time
NGS: Next-generation sequencing
MCH: Mean cell hemoglobin
MCV: Mean cell volume
pg: Picogram
fl: Femtoliter
gDNA: Genomic DNA
SNVs: Single nucleotide variations
indels: Insertions and deletions
