# Supplementary material for: Third-generation sequencing identified a novel complex variant in a patient with rare alpha-thalassemia
Source: BMC Pediatr. 2024 May 13;24:330. doi: 10.1186/s12887-024-04811-1 (PMC11089783; doi:10.1186/s12887-024-04811-1)
Supplement: Supplementary file 1 — Supplementary Material 1 [file 12887_2024_4811_MOESM1_ESM.doc]

**
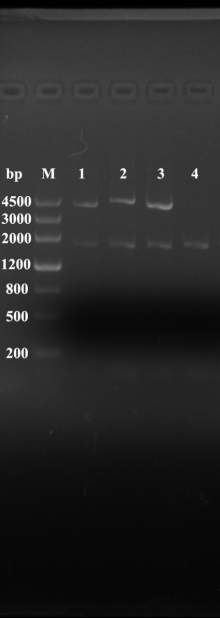
**

**Figure S1.** The original images of PCR-electrophoresis (performed to confirme ααα^anti3.7^). "M" was the electrophoresis image of "marker". "1" was the image of the "patient" (ααα^anti3.7^). "2" was the image of "positive control with ααα^anti4.2^". "3" was the image of "positive control with ααα^anti3.7^". "4" was the image of "negative control".

α Gel


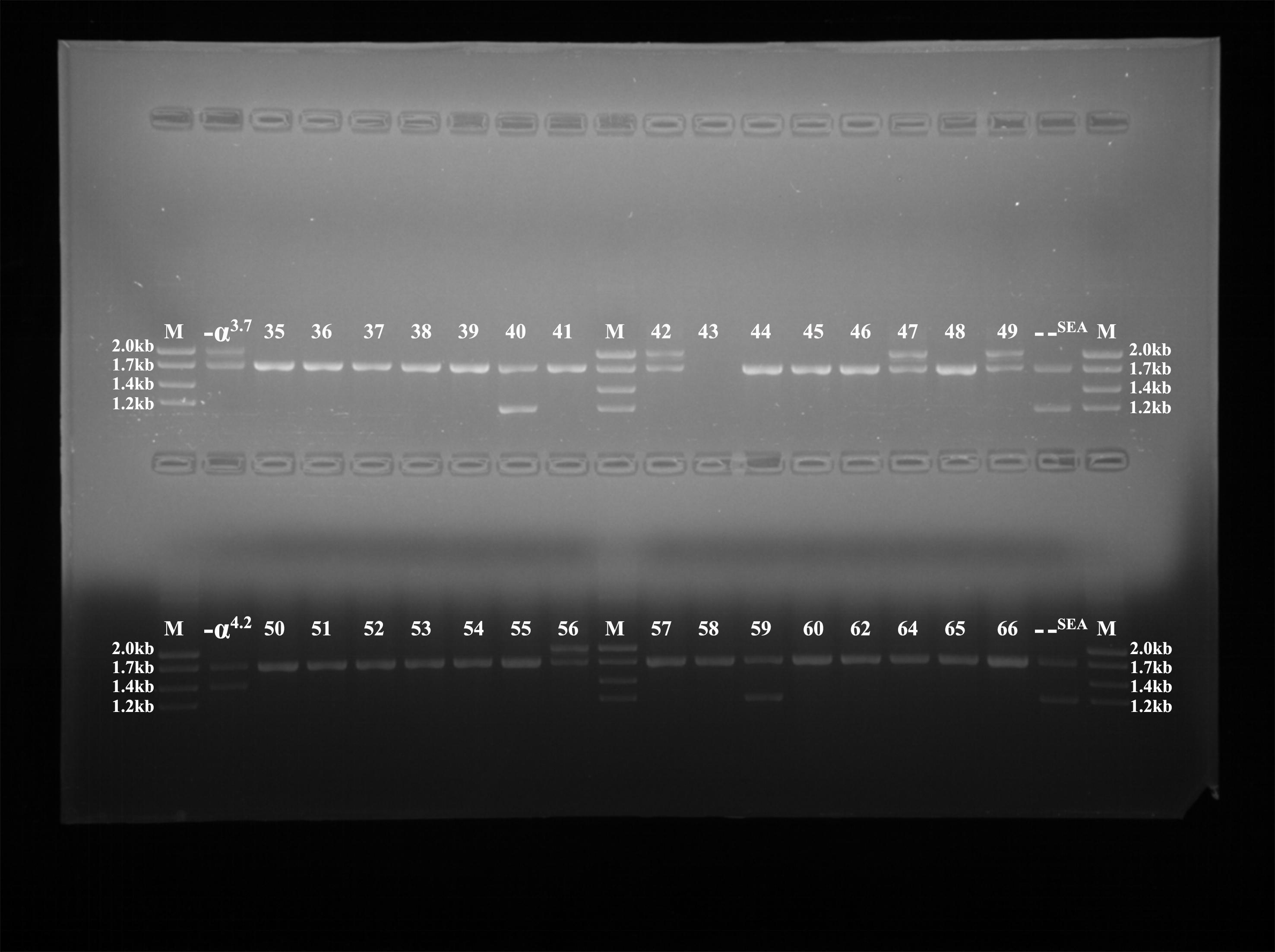


**Figure S2.** The original images of **Figure 1A** (gap polymerase chain reaction performed to detect 3 types of common deletions in a-thalassemia). "M" was the electrophoresis image of "marker". "56" was the image of the "patient" with -α^3.7^, "57" was the image of "negative control (NC)", "-α^3.7^" was the image of "positive control (PC)".

α Mem1


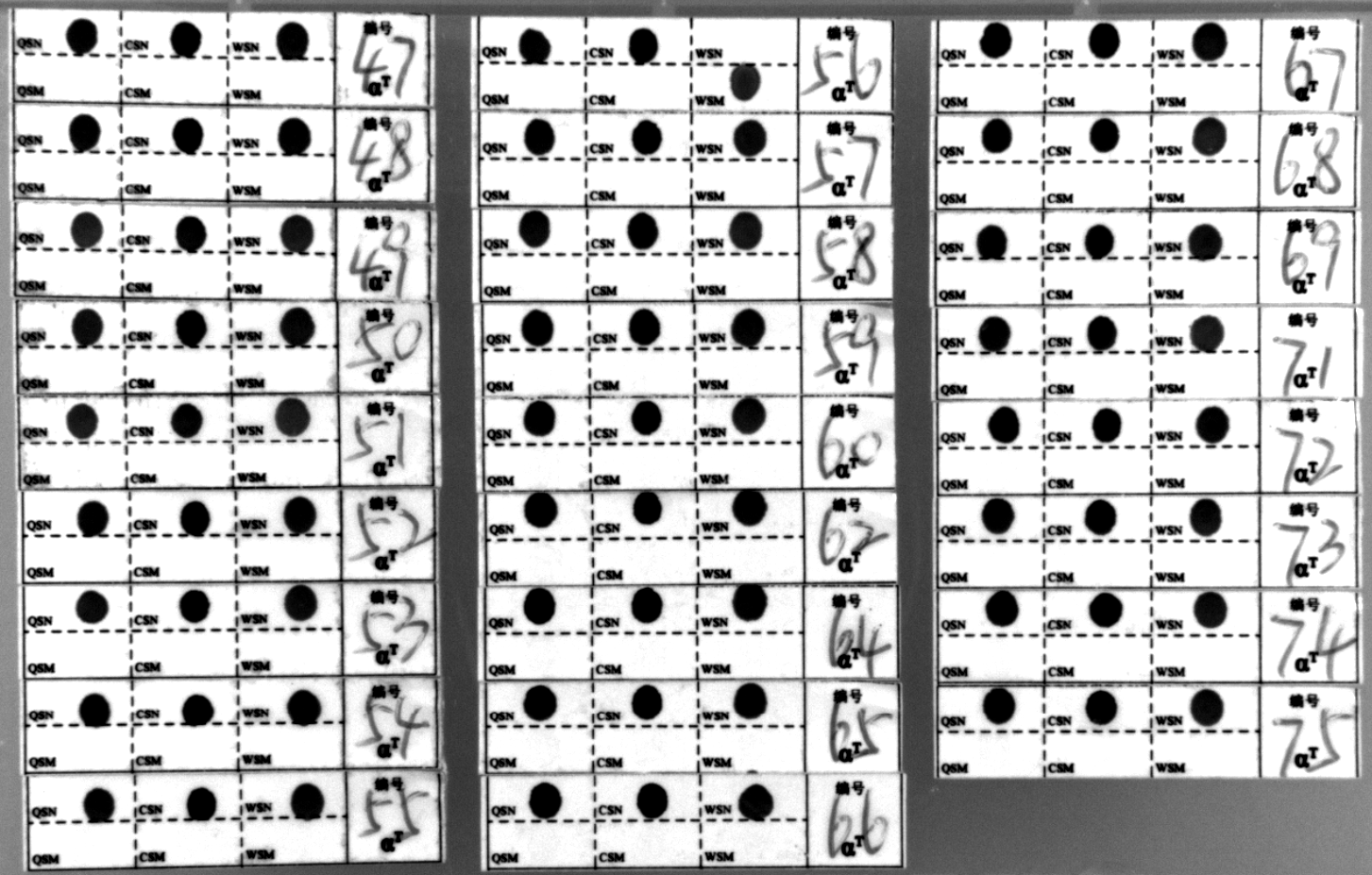


**Figure S3.** The original images of **Figure 1B** (PCR reverse dot blot, PCR-RDB, which performed to detect 3 types of common variants in a-thalassemia). "56" was the image of the "patient" with homozygous Hb Westmead variation, "57" was the image of "negative control (NC)".

α Mem2


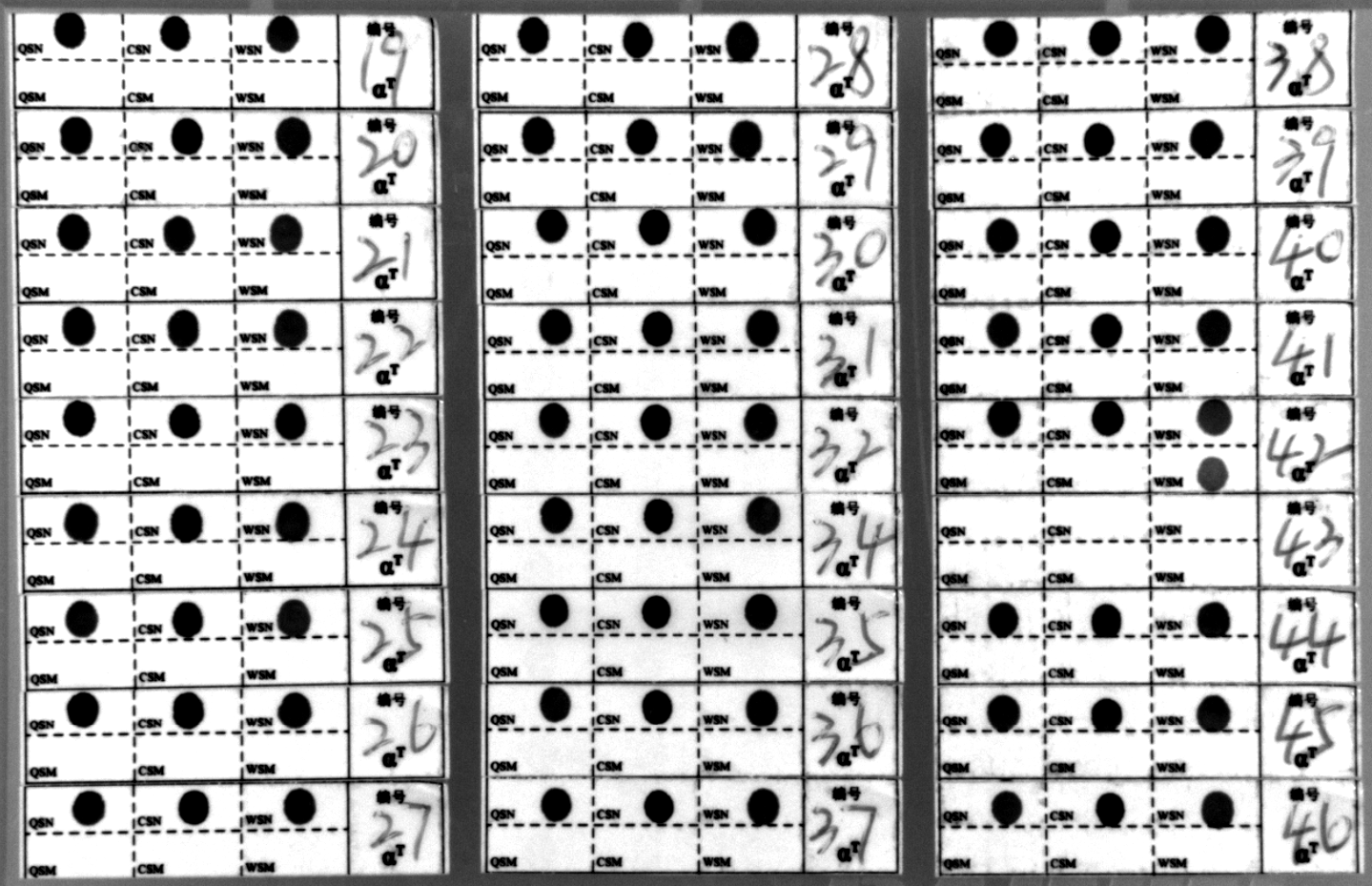


**Figure S4.** The original images of **Figure 1B** (PCR-RDB performed to detect 3 types of common variants in a-thalassemia). "42" was the image of "positive control (PC)" with heterozygous Hb Westmead variation.

β Mem1


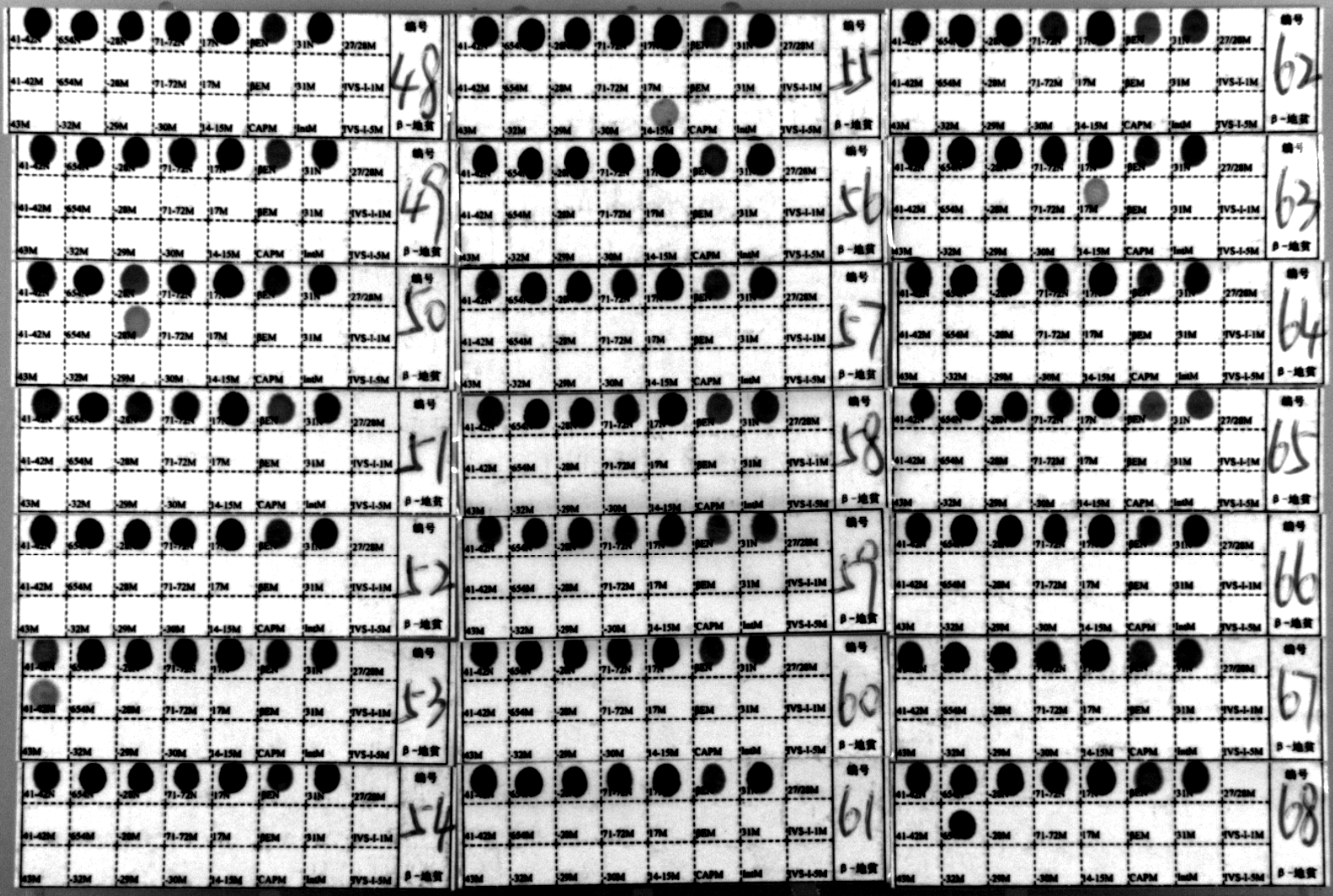


**Figure S5.** The original images of **Figure 1B** (PCR-RDB performed to detect 17 types of common variation in β-thalassemia). "56" was the image of the "patient" without variant, "57" was the image of "negative control (NC)", "68" was the image of "positive control (PC)" with heterozygous c.316-197C > T [IVS-II-654(C→T)].
